# Mechanism of action of Bioactive Endodontic Materials

**DOI:** 10.1590/0103-6440202305278

**Published:** 2023-03-06

**Authors:** Carlos Estrela, Luciano Tavares Angelo Cintra, Marco Antônio Hungaro Duarte, Giampiero Rossi-Fedele, Giulio Gavini, Manoel Damião Sousa-Neto

**Affiliations:** 1 School of Dentistry, Federal University of Goiás, Goiânia, GO, Brazil; 2 School of Dentistry, São Paulo State University (Unesp), Araçatuba, SP, Brazil; 3 School of Dentistry, Bauru, SP, University of São Paulo, SP, Brazil; 4 Adelaide Dental School, University of Adelaide, Adelaide, SA, Australia; 5 School of Dentistry, University of São Paulo, São Paulo, SP, Brazil; 6 School of Dentistry, University of São Paulo, Ribeirão Preto, SP, Brazil

**Keywords:** Bioactive materials, calcium hydroxide, calcium silicate cement mineral trioxide aggregate, Portland cement

## Abstract

A continuous search for bioactive materials capable of supporting the replacement of damaged pulp tissue, with effective sealing potential and biocompatibility, has represented the attention of studies over the last decades. This study involves a narrative review of the literature developed by searching representative research in PUBMED/MEDLINE and searches in textbooks associated with the mechanism of action of bioactive materials (calcium hydroxide, mineral trioxide aggregate (MTA), and calcium silicate cements). The reflective analysis of the particularities of the chemical elements of these materials, considering the tissue and antibacterial mechanism of action, allows a better understanding of the characteristics and similarities in their tissue responses. Calcium hydroxide paste remains the antibacterial substance of choice as intracanal dressing for the treatment of root canal system infections. Calcium silicate cements, including MTA, show a favorable biological response with the stimulation of mineralized tissue deposition in sealed areas when in contact with connective tissue. This is due to the similarity between the chemical elements, especially ionic dissociation, the potential stimulation of enzymes in tissues, and the contribution towards an alkaline environment due to the pH of these materials. The behavior of bioactive materials, especially MTA and the new calcium silicate cements in the biological sealing activity, has been shown to be effective. Contemporary endodontics has access to bioactive materials with similar properties, which can stimulate a biological seal in lateral and furcation root perforations, root-end fillings and root fillings, pulp capping, pulpotomy, apexification, and regenerative endodontic procedures, in addition to other clinical conditions.

## Introduction

Root canal treatment (RCT) aims to restore the health of damaged pulp and periapical tissues, by eliminating the agents responsible for inflammation and infection ^1^. Antibacterial strategies have been suggested for infected root canal decontamination, including root canal preparation (emptying and enlargement), irrigation protocols, intracanal dressing, endodontic and coronal sealing. The anatomical complexity and the microenvironment present in pulpal infections make the antimicrobial procedure complex, making the appropriate sanitization process of the root canal system challenging ^1^,^2^.

The endodontic history encompasses the several stages of root canal therapeutic procedures, characterizing the differences observed over time of various endodontic materials proposed. Among the available endodontic materials are included those indicated for the treatment of the inflamed dental pulp, root infections decontamination, treatment of the consequences of traumatic dental injuries (root resorption), apexification, regenerative endodontic procedures, as well as the sealing of root perforations, root-end fillings and root canal filling materials. In this sense, a growing search for bioactive materials and for application in endodontics can be observed ^3^-^100^.

Among the materials well studied in endodontics clinical practice, calcium hydroxide, mineral trioxide aggregate, and new calcium silicate cement can be highlighted ^1^-^33^. One of the basic principles for the selection of material is related to the benefits of its physicochemical, biological, and antimicrobial properties. The material that presents a larger number of these favorable properties would certainly contribute more towards the therapeutic process ^33^,^34^.

Calcium hydroxide was described for application in dentistry by Hermann in 1920 ^4^, and remains a material indicated for the management of root canal infections, apexification, regenerative endodontic treatment, root resorption, etc. The biological behavior of this material constitutes one of the important factors to its therapeutic indications ^4^,^5^,^6^,^33^,^34^,^35^.

Mineral trioxide aggregate (MTA), proposed by Torabinejad (Loma Linda University)^7^ is another well-accepted and widely studied material in endodontics, being initially indicated for the sealing root perforations and retro-fillings. This material is a calcium silicate cement, whose composition is Portland cement (tri- and dicalcium silicate), with the addition of 20% bismuth oxide ^7^,^8^,^9^,^10^,^11^,^12^,^13^,^14^.

Based on the premise and function of improving the physical-chemical properties of this material, with the addition of plasticizing agents, in the following years, different calcium silicate cements (IRoot SP, Endosequence BC sealer, BioRoot™ RCS, Bio Aggregate, calcium-enriched mixture (CEM) among others) were introduced on the dental materials market (Chart 1)^23^,^24^,^25^,^26^,^27^,^29^,^31^.

These materials have been used in various clinical conditions in which one of the expected intrinsic features has been their bioactive potential. The International Organization for Standardization (ISO 10993-1:2018) ^32^ defines biocompatibility as “the ability of a medical device or material to perform with an appropriate host response in a specific application”.

In this sense, the present study reviewed and contextualized some similarities in the mechanism of action of these bioactive endodontic materials (calcium hydroxide, mineral trioxide aggregate, and the new calcium silicate cements)(Box 1), as well as the physicochemical properties of calcium silicate hydraulic cements.

**Box 1:**
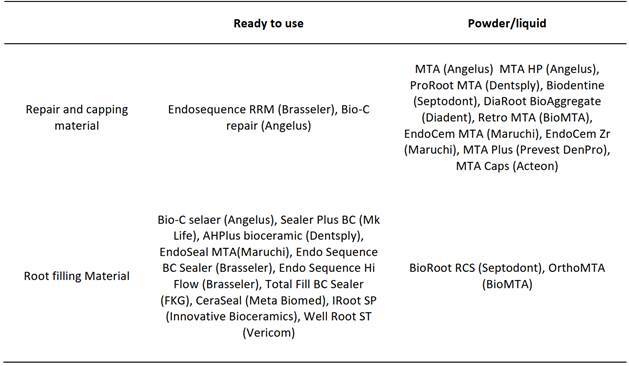
Commercial hydraulic cements, ready to use and powder/liquid, used as repair and root canal filling material.

## Calcium hydroxide

This review of bioactive endodontic materials was structured through previous studies and reviews in which the focus of attention was a reflective analysis and a relationship of the chemical components and mechanisms of action of all these substances.

Calcium hydroxide constitutes a strong base (pH 12.6, slightly soluble in water), which through calcination of calcium carbonate transforms it into calcium oxide. In turn, calcium hydroxide is obtained through the hydration of calcium oxide and when it reacts with carbon dioxide it leads to the formation of calcium carbonate ^6^,^36^.

The properties of calcium hydroxide derive from its ionic dissociation into calcium ions and hydroxyl ions ^5^,^6^, and the action of these ions on bacteria and tissues explains its antimicrobial and biological properties. The ionic release of the calcium hydroxide paste relates to the release of calcium and hydroxyl ions, considering the molecular weight of the calcium hydroxide paste (74.08g). Based on the rule of three, in calcium hydroxide, the ratio of hydroxyl ions and calcium ions is 45.89% and 54.11% ^6^,^33^,^34^,^35^.

Calcium hydroxide potential to stimulate the formation of mineralized tissue (hard tissue barrier) from its ionic dissociation and the biological mechanism of action was described by Holland ^5^. The morphological and immunohistochemical changes observed in the repair process after pulpotomy and direct pulp protection with calcium hydroxide are due to the ionic dissociation of this compound into calcium and hydroxyl ions. This mechanism is strongly accepted due to the ability of hydroxyl ions to produce protein denaturation due to their high pH. The depth of this protein denaturation varies according to the form of calcium hydroxide used (powder, water-soluble paste, or cement) and depend on the vehicle used. These factors are responsible for the amount and speed of formation of hydroxyl ions. In addition to the hydroxyl ions, calcium ions penetrate, which, at the boundary between denatured tissue and living tissue, precipitate in the form of calcium carbonate (reaction of calcium ions with tissue carbon dioxide), being responsible for calcium carbonate granulations, which are birefringent to polarized light. Calcium-protein complexes are also observed below these amorphous granulations of calcium salts, characterizing an area of dystrophic calcification. Thus, in the morphological and histochemical analysis of the pulp repair process after the use of calcium hydroxide, the following zones are characterized: zone of coagulation necrosis (corresponding to the area of protein denaturation of the pulp tissue); superficial granular zone (consisting of coarse granulations of calcium carbonate); deep granular zone (displays fine granulations of calcium salts and represents an area of dystrophic calcification). At 30 days, the repair is complete, and the mineralized barrier is present. The hard tissue barrier formed is composed of the layers (calcium carbonate granulations, dystrophic calcification area, and dentin); cell proliferation zone; and normal pulp zone. Therefore, totaling five zones upon healing is accomplished.

In addition, calcium ions actively participate in the repair process ^5^. Seux et al. ^37^ confirmed these results. Granulations of calcite and fibronectin (glycoprotein) can be an initial stimulus in the formation of a hard tissue barrier. Mizuno & Banzai ^38^ analyzed the effect of calcium ions in dental pulp cells treated with high concentrations of calcium or magnesium ions, and the measurement of fibronectin gene expression. Fibronectin gene expression was stimulated by calcium ions in a dose-dependent manner. Magnesium ions did not influence fibronectin gene expression ^38^. Calcium ions released from calcium hydroxide stimulate fibronectin synthesis in dental pulp cells. Fibronectin can induce the differentiation of dental pulp cells into mineralized tissue forming cells, which are the main cells to form dentinal bridges. Alkaline phosphatase, a hydrolytic enzyme is thought to act by releasing inorganic phosphate from phosphate esters. It is believed to be related to the mineralization process ^39^-^43^, as this enzyme can separate the phosphoric esters to release phosphate ions, which remain free, and react with calcium ions (from the bloodstream) to form a precipitate in the organic matrix, calcium phosphate, which is the molecular unit of hydroxyapatite ^43^. Calcium hydroxide can activate alkaline phosphatase from its high pH, which can initiate or favor the mineralization process ^44^,^45^,^46^.

 According to Holland et al. ^47^, calcium hydroxide and MTA showed similar tissue reactions when inserted into dentin tubes and implanted in rat connective tissue. It was observed Von Kossa-positive granules, birefringent to polarized light in the MTA group. Next to these granulations, there was also irregular tissue like a bridge that was Von Kossa-positive. The dentin walls of the tube exhibited in the tubules a structure highly birefringent to polarized light, usually like a layer and at different depths. In the calcium hydroxide group similar results were observed ^47^. Thus, the mechanism of action of MTA and calcium hydroxide, supporting hard tissue deposition, appear to be similar.

In addition to the biological mechanism of action of calcium hydroxide in connective tissue, its antibacterial mechanism of action previously described ^6^,^62^ should be considered. The pH gradient that occurs at the level of the cytoplasmic membrane of bacterial cells is associated with the production of energy to transport nutrients and organic components into the cell. Complex physiological reactions occur when the pH gradient at the membrane level is affected, influencing chemical transport. In this sense, depending on the pH of the medium, there will be an increase in the availability of nutrients, and an intense transfer that can induce inhibition and toxic effects in the cell. The enzymatic activity of the bacteria is inhibited under conditions of high pH (high concentration of hydroxyl ions) ^6^,^33^,^49^. Thus, chemical transport across the cell membrane is altered by the number of hydroxyl ions present, through the process of lipid peroxidation ^50^. The loss of membrane integrity can be observed through the destruction of unsaturated fatty acids or phospholipids ^50^.

The mechanism of action of calcium hydroxide is associated with the effect of pH on bacterial cell growth, metabolism, and division ^6^,^33^,^51^. The essential enzyme systems of the bacterial cell develop at the level of the cytoplasmic membrane, where they are involved in the last stages of cell wall formation, participate in the biosynthesis of lipids, being responsible for the transport of electrons, as enzymes involved in the process of oxidative phosphorylation. The cytoplasmic membrane is formed by a double layer of phospholipoprotein, that acts as an osmotic barrier for ionized substances and large molecules, whilst being freely permeable to sodium ions and amino acids (selective permeability)^6^,^33^,^49^,^52^,^53^,^54^,^55^,^56^.

The biological effect of pH on bacterial enzyme activity influences cell metabolism, growth, and division. The high pH of calcium hydroxide (12.6), and the release of a high amount of hydroxyl ions, alters the integrity of the cytoplasmic membrane through chemical aggressions to organic components and nutrient transport, or through the destruction of phospholipids or unsaturated fatty acids from the cytoplasmic membrane (lipid peroxidation process - saponification reaction) ^6^,^33^,^49^,^50^.

The explanation of the mechanism of action of calcium hydroxide in the control of bacterial enzymatic activity leads to the hypothesis of an irreversible bacterial enzymatic inactivation (under extreme pH conditions, during prolonged periods), and reversible bacterial enzymatic inactivation; enzymatic action is reestablished if the ideal pH returns, with a subsequent return to normal activity ^6^,^52^,^55^,^56^,^62^. Irreversible enzyme inactivation can be demonstrated from a direct antibacterial action of calcium hydroxide on bacteria ^55^. Reversible enzymatic inactivation can be observed from an indirect action ^56^ when the bacteria are inside the dentinal tubules and the intracanal dressing needs dissociation and diffusion for action at a distance (indirect action) ^6^,^33^,^55^-^57^,^62^. In this case, the length of time of the intracanal dressing remains crucial ^55^,^56^,^57^. The hydroxyl ions of calcium hydroxide can hydrolyze the Lipopolysaccharide (LPS) present in the cell wall of Gram-negative bacteria, degrading lipid A and neutralizing its residual effect after cell lysis. Neutralization of bacterial toxins is an essential aspect in the selection of an antimicrobial agent ^58^,^59^. Khan et al. ^60^ evaluated the effect of calcium hydroxide on pro-inflammatory cytokines and neuropeptides. The hypothesis that calcium hydroxide reduces levels of the inflammatory mediators IL-1α, TNFα and Calcitonin Gene-related Peptide (CGRP) has been tested. The results indicate that calcium hydroxide denatures IL-1α, TNFα, and CGRP and that denaturation of these proinflammatory mediators is a potential mechanism that contributes to the resolution of apical periodontitis. The results of long-term calcium hydroxide treatment of teeth with pulp necrosis and apical periodontitis were analyzed by Best et al. ^61^ in a retrospective cohort study. Teeth treated with calcium hydroxide were evaluated using a standardized protocol and re-evaluated over a 3 months period until radiographic healing was observed. Pre and postoperative periapical radiographs were evaluated using the PAI system. Of the 242 cases, 219 participants completed their treatment with an annual follow-up. The median time of calcium hydroxide dressing was 5.4 months with a range of 1 to 12 months. Overall, at the last follow-up visit, 90.0% (197/219) were classified as “healed”. Long-term calcium hydroxide in the treatment of teeth with pulp necrosis and apical periodontitis resulted in a highly predictable outcome, and there was no association between long-term use of calcium hydroxide and fracture incidence. Therefore, calcium hydroxide is a suitable material as an intracanal dressing for teeth diagnosed with pulp necrosis and apical periodontitis.

In the dentistry market, several pastes containing calcium hydroxide have been commercialized; however, the pure paste Pro-analysis may be favored by having a higher ionic concentration of its chemical elements. It is essential to remember that this material, when applied as intracanal dressing, should only be delivered inside the root canal since it constitutes a very strong base, therefore is toxic.

## Hydraulic cements

Hydraulic cements are materials that depend on water for the occurrence of their hardening. The first calcium-silicate based hydraulic material that emerged for use in dentistry was Mineral Trioxide Aggregate. Then, new materials using tricalcium and dicalcium silicate emerged. The Chart 1 presents the commercial hydraulic cements, ready to use and powder/liquid, used as repair and root canal filling materials.

## Mineral Trioxide Aggregate

Mineral trioxide aggregate (MTA) was incorporated into endodontics for different clinical practice applications ^7^-^32^. This bioactive endodontic cement mainly containing calcium and silicate elements was introduced in the 1990s by Torabinejad and approved by the Food and Drug Administration to be used in the United States in 1997 ^7^. Mineral Trioxide Aggregate is composed of approximately 75% Portland cement, 5% calcium sulfate hydrated (gypsum), and 20% bismuth oxide. The MTA chemical composition include tricalcium silicate, tricalcium aluminate, tricalcium oxide, and silicate oxide ^10^, with tricalcium silicate being its main constituent ^15^,^16^,^21^-^23^,^63^.

Wucherpfenning & Green ^64^ described that MTA and Portland cement have identical macroscopical and microscopical characteristics, and using X-ray diffraction analysis. These materials similarly support matrix formation in cultures of osteoblast-like cells, and also the apposition of reparative dentin when used as direct pulp capping material in rat teeth. Estrela et al. ^65^ chemically evaluated the elements present in MTA and Portland cements by fluorescence spectrometer X-ray. The results showed that Portland cements contain the same chemical elements as MTA except that MTA also contains bismuth. In chemical assays of Portland cement, the components found in greater percentages were: CaO (58.5%), SiO_2_ (17.7%), Al_2_O_3_ (4.5%). Based on this chemical similarity between compositions of mineral trioxide aggregate and Portland cement, Holland et al. ^19^ tested the behavior of dog dental pulp after pulpotomy and direct pulp protection with these materials. After pulpotomy, the pulp stumps of 26 roots of dog teeth were protected with MTA or Portland cement. Sixty days after treatment the histomorphological analysis revealed a complete tubular hard tissue bridge in almost all specimens. MTA and Portland cement show similar comparative results when used in direct pulp protection after pulpotomy. These results described above appear coherent since the chemical compositions of MTA and Portland cement are similar.

The properties of MTA have been extensively studied (physically, chemically, and biologically) using different methodologies, and demonstrating good potential to seal lateral and furcal root perforations, root-end fillings, pulp capping, pulpotomy, apexification and regenerative endodontic procedures, and other clinical conditions ^7^-^32^,^63^. Parirokh & Torabinejad ^11^ reviewed the literature analyzing different methodologies involved in the clinical applications of MTA, in animals and humans and synthesized the mechanism of action highlighting it as a bioactive material with the potential to stimulate an ideal environment for healing. MTA activity in direct contact with connective tissue forms calcium hydroxide that releases calcium ions for cell attachment and proliferation; allows an antibacterial environment due to the high pH; modulates cytokine production; stimulates the differentiation and migration of hard tissue producing cells; forms hydroxyapatite (or carbonated apatite) on the surface of the MTA and provides a biological seal.

Since MTA is a calcium silicate cement, new materials with similar composition have been proposed with additional characteristics that allow an improved clinical application, which facilitate handling and manipulation and minimizes the coronal discoloration. To achieve this, the new calcium silicate cements (also named bioceramics) form a colloidal structure after hydration and sequentially develop into a hard structure ^23^.

The advantages that have been described in the literature of the new calcium silicate cements are related to their physicochemical and biological properties, including excellent sealing potential, due to their physicochemical interaction with the local environment, and high biocompatibility ^66^. Furthermore, they have high compressive strength and dentin-like physical characteristics ^66^,^67^. Their sealing ability results from their interaction with dentin and the formation of a mineralized intermediate zone, with tag-like structures that extend into the dentinal tubules and, thus, they act as a micromechanical anchorage to the dentin ^68^,^69^. Another characteristic responsible for the good sealability of bioceramic cements relates to their expansion after hydration and setting ^70^.

## Calcium Silicate Cements

Tissue reactions against calcium silicate cements begin before the material sets and continue until complete tissue repair. The initial reactions are triggered by the hydration of di and tri-calcium silicate ^71^, favoring the dissolution of ions from the anhydrous material ^23^. In this first step, the formation of calcium silicate hydrate and calcium hydroxide ^72^ occurs, resulting in the crystallization of the hydrates that determines the strength of the material ^73^. This hydration can occur through contact with water or liquids containing water ^73^, such as the fluid of living tissues ^47^. From the formation of calcium hydroxide and its dissociation, there is a continuous release of calcium and hydroxyl ions ^74^, providing an alkaline environment conducive to the formation of mineralized tissue ^47^,^75^,^76^. The alkaline medium provides an unfavorable environment for bacterial growth, resulting in the antimicrobial activity of this material ^65^. In addition, this alkalinity promotes moderate tissue damage through protein denaturation, thus activating alkaline phosphatase, an enzyme that stimulates the release of inorganic phosphate from phosphate esters ^72^. Alkaline phosphatase works by separating phosphoric esters and releasing phosphate ions. Calcium ions react with free phosphate ions, resulting in the formation of calcium phosphate ^77^, the main component of hydroxyapatite. These calcium phosphate crystal structures function as the initial matrix for mineralization ^47^,^78^-^80^. Calcium ions also react with the carbon dioxide present in the tissue, forming a precipitate, calcium carbonate, or granulated calcite ^47^. In connective tissue, it is possible to visualize these granulations as they are birefringent to polarized light ^79^,^81^. Adjacent to the granulations, fibronectin begins to accumulate, leading to the formation of dystrophic calcifications. Therefore, calcium ions also participate in cell signaling for cell proliferation and the production of proteins that participate in the mineralization process ^82^.

During the tissue repair process promoted by calcium silicate cements, as described previously and similarly to the response to calcium hydroxide, it is possible to distinguish 5 different zones: 1. zone of necrosis by coagulation, corresponding to the area of protein denaturation; 2. superficial granular zone, composed of more robust granulations of calcium carbonate; 3. deep granular zone, composed of granulations of calcium salts and an area of dystrophic calcification; 4. zone of cell proliferation, composed of young cells in activity; 5. repaired or normal tissue zone.

## Physical-chemical properties of calcium-silicate based hydraulic cements

Among the physical properties that a root canal filling and repair materials should present are setting, solubility, flow and adequate radiopacity ^10^,^83^,^84^.

Hydraulic cements that have as their main component the di and tri calcium silicates are available in the ready-to-use form or powder and liquid form. In both forms of presentation, water is responsible for providing the setting reaction of these materials. In the case of ready-to-use water comes from dentinal tubular fluids and periapical tissues, while in the powder/liquid form, water is the main component of the liquid (Chart 1).

About the setting time, some substances can slow down or accelerate the setting time ^85^. In the composition of ProRoot MTA, there is 5% dihydrate calcium sulfate, which is a setting retarder, making this reaction require 2 hours and 57 minutes ^10^. Other MTA formulations emerged in which calcium sulfate was removed and consequently there was a reduction in setting time, reaching 15 to 40 minutes ^85^,^86^. In ready for use forms, due to the need for water coming from the environment, a significant question is whether the setting occurs entirely. Studies following ISO 6876/2012 have shown that setting occurs between 4 and 9 hours ^87^,^88^. There are formulations that present calcium chloride as a setting accelerator ^85^. The presence of this additive in the composition of hydraulic cements makes the setting occur rapidly and can reduce the setting time to 1 hour ^85^.

Regarding solubility, laboratory studies show great variability that may also vary according to the solution in which the material was immersed in, distilled water or phosphate buffer solution. But most studies show that solubility values transcend what is recommended by the ISO standard that recommends not being greater than 3% ^84^,^87^,^88^. Studies that analyzed the ready-to-use forms observed values of solubility above 10%, according to the brand; an occurrence that may favor the presence of voids in root canal fillings ^87^-^89^.

In performing endodontic root canal fillings, the flow is a property that will provide the ability for the material to penetrate areas of morphological complexity. The literature shows that the flow of hydraulic materials is in accordance with the ISO standard, and with values higher than epoxy resin materials such as AH Plus ^87^,^88^.

Radiopacity is the property that allows the material to be discerned against the mineralized structures of the tooth and the jaws. The first hydraulic cements used bismuth oxide as a radiopacifier agent ^10^; however, this substance causes greater porosity to the material, besides promoting the darkening of the dental hard tissues ^90^-^95^. The bismuth oxide is reduced in bismite, which has dark gray color, and collagen has an affinity to this substance ^92^. To avoid darkening with bismuth oxide, it is sufficient to add 5% of zinc oxide to the formulation ^91^. New radiopacifier alternatives were proposed such as zirconium oxide, calcium tungstate, and others ^96^,^97^. Despite presenting lower radiopacity values, when increased in the percentage of 20% or greater, these provide radiopacity above 3mm Al, which is the minimum recommended by the ISO standard ^96^,^97^. Moreover, these radiopacier agents do not lead to the risk of alteration of tooth color ^92^ and do not interfere with the physical chemical properties of hydraulic cement ^86^.

With regard to the chemical properties of hydraulic cements, their setting reaction involves the transformation of di and tricalcium silicates, into hydrated calcium silicate and produces a component that is the Portlandite, which is nothing more than calcium hydroxide. Portlandite is the soluble part of the material, as the hydraulic materials are similar in composition to Portland cement, with most Portland cements producing 13 to 17% calcium hydroxide after setting ^86^. The addition of calcium chloride or propylene glycol has favored an increase in pH and the release of calcium ions ^85^,^98^. Propylene glycol also reduces blood-promoted darkening ^98^.

Considering the physical chemical properties of hydraulic cements overall, perhaps the high solubility should be considered a limitation. Controlled clinical trials need to be conducted to verify the impact of the high solubility demonstrated in laboratory studies on the clinical performance of these materials ^28^,^99^,^100^.

## Clinical Highlights

The present reflective analysis of the particularities of the chemical elements of the bioactive materials discussed in the present review, which considers their tissue and antibacterial mechanism of action, allows a better understanding of the characteristics and similarity in their tissue behavior.

Calcium hydroxide paste remains the antibacterial substance of choice as an intracanal dressing for the treatment of root canal system infections. This is due to the chemical availability of calcium and hydroxyl ions of calcium hydroxide made available for the surrounding tissues, and the bacterial enzymatic inhibition.

Calcium silicate cements, including MTA, show a favorable biological response regarding the stimulation of mineralized tissue deposition in sealed areas when in contact with connective tissue. This is due to the similarity between the chemical elements, especially to the ionic dissociation, the potential stimulation of tissue enzymes, and the contribution of an alkaline environment due to the pH of these materials.

Bioactive materials, especially MTA and the new calcium silicate cements are effective to support a biological seal. Contemporary endodontics practice has access to bioactive materials with similar properties capable of stimulating biological sealing in lateral and furcation root perforations, root-end fillings and root fillings, pulp capping, pulpotomy, apexification, and regenerative endodontic procedures, in addition to other clinical conditions. The positive impact of these bioactive materials used in therapeutic procedures for various conditions in endodontic clinical practice was to induce a healing response in the injured host tissue and prevent tooth loss and its disastrous consequences.

However, even with the knowledge regarding the materials discussed here, it is important to say that all new material must be deeply studied. The new materials must also undergo long-term clinical tests to verify whether new components added to their formulas, in an attempt to improve some of their properties, may have harmed another property. Further studies are recommended to better understand the clinical translation of the increased solubility demonstrated in laboratory studies for calcium silicate hydraulic cements, as this may compromise their long-term seal, thus potentially increasing the risk of permanence or reoccurrence of apical periodontitis.
